# A study of GafChromic XR Type R film response with reflective‐type densitometers and economical flatbed scanners

**DOI:** 10.1120/jacmp.v4i4.2501

**Published:** 2003-09-01

**Authors:** G. Thomas, R. Y. L. Chu, Frank Rabe

**Affiliations:** ^1^ Department of Radiological Sciences University of Oklahoma Health Science Center 940 Stanton L. Young Boulevard, Biomedical Sciences Building‐127 Oklahoma City Oklahoma 73190; ^2^ Radiology Services Veterans Affairs Medical Center 921 Northeast Thirteenth Street Oklahoma City Oklahoma 73104

**Keywords:** GafChromic film, scanner, densitometer

## Abstract

The GafChromic XR Type R film is a relatively new product for recording high radiation dose in interventional radiological procedures. Means of measuring the film response were studied in this investigation. Two handheld reflective‐type densitometers of different models were compared in the range of 0–8 Gy. They were found to be in excellent agreement. Five reflective flatbed scanners of different models were compared by a simple preliminary test. Their widely differed performances suggest the need of testing a scanner before using it for dosimetry measurement. A selected scanner was further tested for its ability to measure radiation in the range of 0–8 Gy and for the development of a scanning protocol. This experiment suggested the inclusion of a calibration pattern with known exposures and a black reference step in the scanning of a film in RGB mode. Then the red component of this image should be used for dosimetry computation. This method was compared to the use of a red acetate filter. The latter was demonstrated to be a possible alternative for measurement below 5 Gy and when there is no software ability to split an image into color components.

PACS number(s): 87.66.–a, 87.52.–g

## INTRODUCTION

Radiochromic dye films have been found to offer ease of handling, high reproducibility, and to be quite useful for measuring large radiation doses.^1^ ^–^ ^3^ Specifically, the GafChromic dosimetry media (International Specialty Products, Wayne, NJ) has been very useful for the mapping of dose distributions in radiation therapy.^4^ ^–^ ^7^ Increasing concern for the potentially high radiation dose in interventional radiological procedures has led to the use of radiochromic films in this imaging modality.^8^ The GafChromic XR Type R Dosimetry film (International Specialty Products, Wayne, NJ) has recently been introduced to meet this need. Handheld reflective‐type densitometers can be used for spot measurement of these films. In this investigation, two models were compared for their responses to the GafChromic film. For complete coverage of the exposed area, a scanner would be required. For this purpose, five economical flatbed scanners were compared in a preliminary test. One was selected for further study in the development of a scan protocol and method of image analysis.

**Table I:** Characteristics of the scanners tested.

	Microtek ScanMaker E6	Microtek ScanMaker 4800	Umax Mirage IIse	HP ScanJet 4C	Canon LIDE 20
Scanner type	Reflective, flatbed	Reflective, flatbed	Reflective, flatbed	Reflective, flatbed	Reflective, flatbed
Optical resolution	600×1200 dpi	2400×1200 dpi	700×1400 dpi	600×1200 dpi	600×1200 dpi
Color/Bit depth	30‐bit	48‐bit	36‐bit	30‐bit	48‐bit
Light source type	Daylight Fluorescent lamp	Cold‐Cathode Lamp	Cold‐Cathode Lamp	Daylight Fluorescent Lamp	Three‐color RGB LEDs
Light source wavelength	Broadband	Broadband	Broadband	Broadband	Broadband

## >METHODS

### A. Uniformity and film response

A Tobias Associates RPB densitometer (Tobias Associates, Ivyland, PA, on loan from International Specialty Products), which had been modified by the manufacturer and International Specialty Products, was used to read 15 evenly spaced locations of an unexposed GafChromic film (12 cm× 21 cm). This measurement of uniformity was repeated with a Gretag‐Macbeth D19C densitometer (Gretag Macbeth, New Berlin, WI) in the cyan color channel.

**Figure 1:**
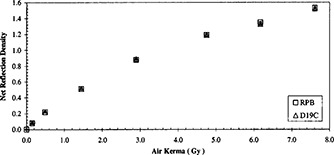
Net reflection densities of a calibration pattern measured by the Tobias RPB and the Gretag D19C Densitometers. The radiation was given at 75 kVp and a half value layer of 5.25 mm of aluminum.

**Figure 2:**
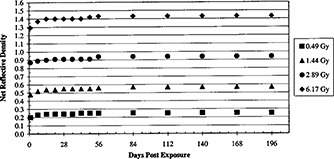
Net reflective densities at 1 day to 196 days after exposure.

To study the film response, an x‐ray machine (Philips Optimus V5000, Philips Medical Systems, Andover, MA) was used to create a calibration tablet. Pieces of the GafChromic film were exposed to different amounts of radiation with a maximum air kerma of 7.61 Gy. The amount of radiation in air kerma was determined by an ion chamber placed free in air (i.e. without backscatter). The beam quality was 75 kVp and a half value layer of 5.25 mm of aluminum. Twenty‐four hours after the exposure, the calibration pattern was read by both densitometers (Fig. 1). In addition, the Tobias RPB densitometer was used to read the calibration tablet once a week for two months and then monthly thereafter to monitor changes in film density (Fig. 2). This tablet was stored in the dark under normal room temperature and humidity, except when it was read.

### B. Selection of a scanner

A radiographic machine (Philip Optimus X‐ray Unit, Philips Medical Systems, Andover, MA) was used to create an image of an aluminum step‐wedge (a maximum thickness of 2.54 cm divided into sixteen steps). The source to the GafChromic film was 101.6 cm. The total air kerma at the film plane was 1.8 Gy with a technique of 125 kVp and half value layer of 4.75 mm of aluminum.

This created tablet was then scanned by five flatbed reflective‐type scanners: Microtek ScanMaker E6, Microtek ScanMaker 4800, Umax Mirage IIse, HP ScanJet 4C, and Canon LIDE 20. Each scanner's characteristics are located in Table I. Each scanner was allowed to warm up for 30 min. The scanning parameters were: RGB mode, 300 dpi, and 25% scaling of the image (i.e. the color image was reduced to 25% of it's original height and width). No color correction factors or filters were used. The lids of all these scanners provided a white color backing to the tablet. To test the reproducibility of measurement, the film was scanned five consecutive times in one day.

**Figure 3:**
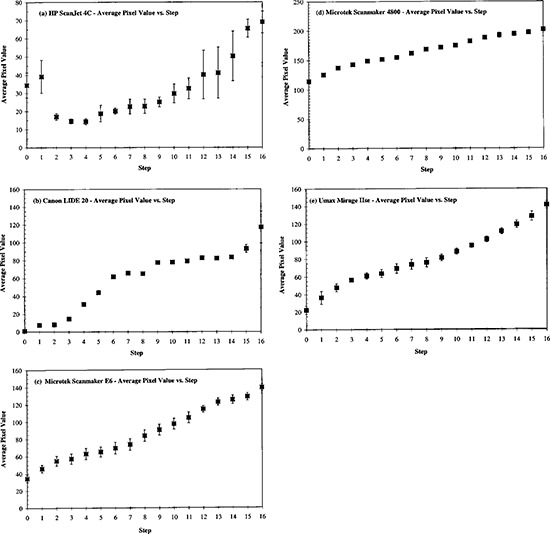
Mean pixel value of five measurements versus step number of an aluminum step wedge. The standard deviations are represented by error bars. Each increment in step number corresponds to an additional thickness of 0.16 cm.

In this investigation, the scanned images were analyzed with the Osiris computer program (University Hospital of Geneva, Geneva, Switzerland). A region‐of‐interest of 55 pixels by 35 pixels was placed at the center of each step to compute the mean pixel value and its standard deviation. These measurements are presented in Fig. 3 for the comparison of the scanners. An examination of these results suggested that the Microtek ScanMaker 4800 to be among the most suitable for the purpose of this project. This scanner was also more accessible for the investigators and was therefore selected for further study.

### C. Performance characteristics of the Microtek ScanMaker 4800

To test the spatial uniformity of this scanner, a 21.5 cm×27.9 cm sheet of unexposed GafChromic film was scanned with the same parameters in the previous section. The resultant image was divided into 32 equal segments (region of interest of 634 pixels by 397 pixels) for analysis with the Osiris computer program.

When Alva *et al*.^9^ investigated the use of a reflective scanner for double‐sided radiochromic films, they compared white backing to black backing, split image into color components, and explored the means of normalizing the color scale. In this present experiment, the GafChromic XR Type R film is single sided with its own reflective paper backing. Nevertheless, all of the above three tests were repeated. A 21.5 cm×27.9 cm sheet of unexposed GafChromic film was scanned in RGB mode with a white backing (inner surface of the lid) and then a black backing. For the black backing, a piece of black glossy paper was cut to the size of the film and placed directly behind it. As with the uniformity test, the resulted image was divided into 32 equal segments (region of interest of 634 pixels by 397 pixels) for analysis with the Osiris Computer Program. The black backing was chosen for subsequent measurements.

**Figure 4:**
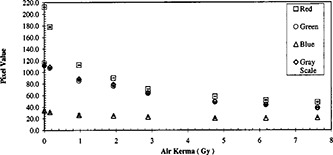
Comparison of responses in gray‐scale mode and color components in the RGB mode for the calibration tablet scanned with the black reference step.

**Figure 5:**
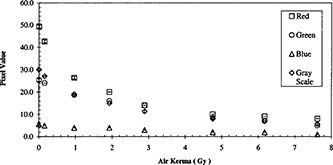
Comparison of responses in gray‐scale mode and color components in the RGB mode for the calibration tablet scanned with a white and black reference step.

**Figure 6:**
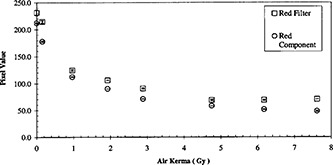
Comparison of the response of the calibration tablets scanned with an external red filter and scanned in RGB mode.

The calibration tablet and a small strip of black paper were placed side‐by‐side and then scanned. Then a small strip of white paper was added and the scan was repeated. Jasc Paint Shop Pro 7 (Jasc Software, Eden Prairie, MN) computer program was used to split these images into red, green, and blue components (Figs. 4 and 5). Scanning in gray‐scale mode was performed for comparison (Figs. 4 and 5). To examine whether a filter can be used as an alternative to the software for color splitting, a piece of red filter (Kodak filter No. 25, Barrington, NJ) was placed between the calibration tablet and the scanner for a scan in RGB mode (Fig. 6).

## RESULTS

### A. Uniformity and film‐response

The fifteen evenly spaced spot measurements of the unexposed film by the Tobias RPB densitometer have a standard deviation of 0.3% in reflection density. The corresponding set of measurements by the Gretag‐Macbeth densitometer has a standard deviation of 0.9%. That is this film, which was used later to test the spatial response of a flat bed scanner, has a uniformity better than 1%.

The film response as measured by the Tobias RPB densitometer and from the Gretag Macbeth densitometer is presented in Fig. 1. Results from both instruments were in agreement throughout the range of air kerma tested.

Changes in the film with time post‐exposure are described in Fig. 2. Representative values at 14, 28, 56, and 112 days are presented in Table II. The densities continued to grow after irradiation until about 14 days post exposure when they became stable.

### B. Selection of a scanner

For each step of the aluminum step wedge image, the mean and standard deviation of the five measurements by a scanner were computed. A plot of pixel value versus step number (i.e. versus decreasing amount of radiation exposure) was used as a relative description of the response by a scanner. The HP ScanJet 4C [Fig. 3(a)] scanner exhibited nonlinear behavior at the thinner steps (i.e., higher transmitted exposure). The relative large standard deviations, as represented by the error bars, suggested the worst reproducibility among the five scanners. The Canon LIDE20 [Fig. 3(b)] scanner was more reproducible. Its response curve showed a rather narrow latitude. The slope differed significantly from zero only in the interval of step 3 to step 7 [Fig. 3(b)]. The remaining three scanners, the Microtek E6 ScanMaker [Fig. 3(c)], the Microtek ScanMaker 4800 [Fig. 3(d)], and the Umax Mirage IIse [Fig. 3(e)], responded rather monotonically (i.e. the pixel value increases continually with the step number). Among these three scanners, the measurements by Microtek ScanMaker 4800 had the smallest standard deviations. (The error bars are not distinguishable from the data symbols in the corresponding graph.)

### C. Performance of the Microtek 4800 ScanMaker

For each of the 32 sections of the unexposed film scanned by the Microtek 4800 ScanMaker, the mean pixel value was computed. The results have a range from 96 to 102 and the distribution has a mean of 99±4. The non‐uniformity in the response of the scanner over an area of 21.5 cm by 27.9 cm is therefore about 4%. When the film was scanned with the black color backing, the average pixel reading increased to 120±2. If this meant more reflected light in the scanning, a black color backing would be preferred.

**Table II:** Net reflective densities post exposure. Percent changes from measurements at 24 h are enclosed in parentheses.

Air kerma	Net density at 1 day from exposure	14 days	28 days	56 days	112 days
0.49 Gy	0.22	0.24	0.24	0.25	0.25
		(9.1%)	(9.1%)	(13.6%)	(13.6%)
1.44 Gy	0.51	0.54	0.55	0.56	0.56
		(5.9%)	(7.8%)	(9.8%)	(9.8%)
2.89 Gy	0.88	0.90	0.91	0.94	0.94
		(2.3%)	(3.4%)	(6.4%)	(6.4%)
6.17 Gy	1.33	1.40	1.41	1.43	1.43
		(5.3%)	(6.0%)	(7.5%)	(7.5%)

Figure 4 displays the responses in gray‐scale mode and the color components in the RGB mode for the calibration tablet with the black backing and a black reference step. The blue component shows insensitivity to the varying amount of air kerma. The green component of the RGB scan mode and the gray‐scale scan mode behave similarly. The red component displays a larger range in pixel values.

When the white reference step was included in the scanning, the shapes of the response curves for the gray‐scale mode and the three‐color components changed very little (Fig. 5). However, the magnitudes were reduced fourfold.

The usage of a red color filter in RGB scan mode is compared to the red color component from the RGB mode in Fig. 6. The former yielded larger pixel values (i.e. brighter light intensities). Both response curves decrease with increasing air kerma until about 5 Gy where the former appears to level off. The red color component response curve continues to decrease beyond 5 Gy.

**Figure 7:**
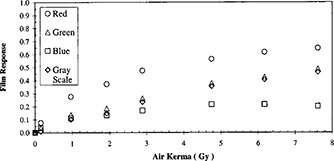
Film response computed as the logarithm of the ratio of color intensities of nonirradiated and irradiated film.

Alva *et al*.^9^ presented their results in derived values. They defined “film response” to be the logarithm of the ratio of color intensity of non‐irradiated film to the diminished color intensity of irradiated film. With this formalism, the data in Fig. 4 was translated to the results in Fig. 7. In comparison to the reflection densities in Fig. 1, these film responses also increase with increasing air kerma. However, their growth rates decrease more rapidly.

## DISCUSSION

Giles and Murphy^8^ demonstrated that the optical density of an exposed GafChromic XR film became fairly stabilized by 24 h post exposure. They monitored the changes in the optical density to about 12 days post‐exposure. International Specialty Products^10^ published similar monitoring of the GafChromic XR Type R film for four days post‐exposure. We extended the monitoring of this product to 196 days (Fig. 2). Measurements of net reflective density at 14 days post exposure with 1.44 Gy can be 5.9% larger than measurements made at 24 h. In the long‐term storage, the net reflective density changes less than 9.8%.

Five reflective flatbed scanners of different models, which were available to the investigators, were compared. These scanners are not meant to be representatives of the respective models. The aluminum step wedge was chosen as a quick and simple screening test for the scanners. The aluminum step wedge image is not equivalent to a calibration pattern of known air kermas.

The findings in this present study of GafChromic XR Type R film with a flatbed scanner are in agreement with the study of Alva *et al*.^9^ for GafChromic MD 55‐2 film in several respects. The red component from the RGB scan mode showed the highest sensitivity. The blue component showed the least sensitivity. The response curve measured in gray‐scale scan mode behaved similarly to the green component in the RGB scan mode. Alva *et al*.^9^ used a black and white reference step to normalize the color in scanning. However, in our study we found increased pixel values when only the black reference step was added to the calibration pattern. The larger range of pixel values might present results in better accuracy.

When a sheet of unexposed XR Type R film was scanned, the color of the backing made a difference in the measured pixel values. This dependence appears to be in agreement with the suggestion of Alva *et al*.^9^ for scanning GafChromic MD 55‐2 film. However, the single emulsion Type R film has its own backing already. The effect and its cause of additional backing materials have not been explored further in this investigation.

Odero *et al*.^11^ had suggested the use of a red acetate filter to improve the sensitivity in measurement. Alva *et al*.^9^ demonstrated the almost complete suppression of the blue color component by a red filter. With these suggestions, we compared the XR type R film response measured by the red component from a scan and the response measured with the insertion of a red color filter in RGB scan mode (Fig. 6). The brighter intensity of the latter could be attributed to the partial transmission of the green color though the red acetate filter.^9^ For readings less than 5 Gy, the two responses are sufficiently similar to suggest that both are acceptable means of measurement.

Measurements with both densitometers were conducted at 24 h post exposure. In the search for the proper scanner and scanning protocol, the scan of the calibration tablet was delayed to more than 14 days post‐exposure. The slight amount of post‐exposure growth (Table I) cannot explain the differences between the response measurements as presented by the two instruments (Figs. 1 and 7). These two methods of measurements could be considered equivalent, but not identical. Presentation of the scanner's results in the derived quantity, “film response” (Fig. 7), allows a comparison with the densitometers and conforms to the formalism of Alva *et al*.^9^ We presented our results in pixel values, which were directly obtainable from a scanner. Such choice allowed expeditious screening of scanners and the correlation of reflected light intensities and scanning conditions.

## CONCLUSION

Means of measuring the film response of the GafChromic XR Type R film were studied in this investigation. The modified RPB densitometer and the D19C reflection densitometer behaved remarkably similarly in the range of air kerma tested. An economical flatbed reflective scanner can be used to measure the two‐dimensional radiation distribution on the film. However, scanners can differ widely in performance. In the testing of a scanner, we would suggest (i) using an image of a step wedge or an air kerma calibration pattern to study the scanner's response curve, and (ii) repeating measurements of this pattern on different days to study the reproducibility of the scanner's performance. In the use of a scanner for dosimetry measurements, it is essential to include a calibration pattern and a black reference step for the normalization of the color scale. The choice of color for the backing of the film can affect the results and therefore should be the same for calibration and dosimetry measurements. The red color component of the RGB scan mode is the optimal choice in measuring film response. In the absence of necessary software to split an image to color components, a red color filter can be used as an alternative. While the means tested in this investigation differ in sensitivity and accuracy at large air kermas, they can be calibrated for measurements below 5 Gy.
